# Association between Antibody Responses to Epstein-Barr Virus Glycoproteins, Neutralization of Infectivity, and the Risk of Nasopharyngeal Carcinoma

**DOI:** 10.1128/mSphere.00901-20

**Published:** 2020-12-02

**Authors:** Qian-Ying Zhu, Xiang-Wei Kong, Cong Sun, Shang-Hang Xie, Allan Hildesheim, Su-Mei Cao, Mu-Sheng Zeng

**Affiliations:** a State Key Laboratory of Oncology in South China, Collaborative Innovation Center for Cancer Medicine, Guangdong Key Laboratory of Nasopharyngeal Carcinoma Diagnosis and Therapy, Sun Yat-sen University Cancer Center (SYSUCC), Guangzhou, China; b State Key Laboratory of Oncology in South China, Department of Cancer Prevention Research, Sun Yat-sen University Cancer Center (SYSUCC), Guangzhou, China; c Division of Cancer Epidemiology and Genetics, National Cancer Institute, Bethesda, Maryland, USA; University of North Carolina, Chapel Hill

**Keywords:** Epstein-Barr virus, nasopharyngeal carcinoma, glycoproteins, antibody, neutralization

## Abstract

While Epstein-Barr virus (EBV) is the major cause of nasopharyngeal carcinoma (NPC), the value of the humoral immune response to EBV glycoproteins and NPC development remains unclear. Correlation between antiglycoprotein antibody levels, neutralization of EBV infectivity, and the risk of NPC requires systematic study. Here, we applied a cytometry-based method and enzyme-linked immunosorbent assay to measure neutralization of infectivity and antibody response to EBV glycoproteins (gH/gL, gB, gp350, and gp42) of plasma samples from 20 NPC cases and 20 high-risk and 20 low-risk healthy controls nested within a screening cohort in Sihui, southern China. We found that NPC cases have similar plasma neutralizing activity in both B cells and epithelial cells and EBV glycoprotein-specific IgA and IgG antibody levels compared with those of healthy controls. Significant correlations were observed between gH/gL IgG and gB IgG and the neutralizing ability against EBV infection of epithelial cells and B cells. These results indicate that a high level of glycoprotein antibodies may favor protection against primary EBV infection, instead of being low-risk biomarkers for NPC in long-term EBV-infected adults. In conclusion, this study provides novel insights into the humoral immune response to EBV infection and NPC development, providing valuable leads for future research that is important for prevention and treatment of EBV-related diseases.

**IMPORTANCE** Epstein-Barr virus (EBV) is a human oncogenic gammaherpesvirus that infects over 90% of humans in the world and is causally associated with a spectrum of epithelial and B-cell malignancies such as nasopharyngeal carcinoma (NPC). A prophylactic vaccine against EBV is called for, but no approved vaccine is available yet. Therefore, EBV remains a major public health concern. To facilitate novel vaccines and therapeutics for NPC, it is of great importance to explore the impact of humoral immune response to EBV glycoproteins before the development of NPC. Therefore, in this study, we systematically assessed the correlation between antiglycoprotein antibody levels, neutralization of EBV infectivity, and the risk of NPC development. These results provide valuable information that will contribute to designing effective prevention and treatment strategies for EBV-related diseases such as NPC.

## Abstract (precis)

Epstein-Barr virus (EBV) is a human oncogenic gammaherpesvirus that infects over 90% of humans in the world and is causally associated with a spectrum of epithelial and B-cell malignancies such as nasopharyngeal carcinoma (NPC). A prophylactic vaccine against EBV is called for, but no approved vaccine is available yet.

## INTRODUCTION

Nasopharyngeal carcinoma (NPC) is a complex tumor involving genetic predisposition, environmental factors, and infection with Epstein-Barr virus (EBV) (1). East and Southeast Asia are high-prevalence areas, accounting for 70% of NPC cases in the world (2). More than 90% of adults globally are estimated to be infected with EBV (3). Malignant transformation of epithelial cells infected with EBV can result in the development of NPC (4). Levels of antibodies targeting EBV proteins, such as EBV capsid antigen (VCA) and EBV nuclear antigen-1 (EBNA1), have been widely used for NPC screening as high-risk biomarkers (5). Considering the EBV exposure at mucosal epithelium, IgA and IgG responses were usually evaluated together (6).

It has been demonstrated that EBV glycoprotein gH/gL binds to EphA2 on the cell surface to facilitate EBV fusion into epithelial cells (7, 8). For EBV infection into B cells, gp350 binds to its receptor protein CR2 and then gH/gL/gp42 trigger the fusion process mediated by gB (9). In order to find out protective biomarkers for NPC, evaluations of antibody against glycoproteins involved in viral entry and the risk of NPC have been conducted. Recent studies showed that higher levels of neutralizing antibody against gp350 represented a lower risk of NPC in high-risk family members but not in the general population (10, 11). The role of antibodies against gp350 and non-gp350 glycoproteins in protection for NPC should be verified in different populations.

There is no licensed EBV vaccine, and previous studies mostly focused on gp350 because of its highest-expression abundance on the viral surface (12). However, results of EBV prophylactic vaccine trials in humans showed that gp350 vaccine could somehow decrease the incidence rate of acute infectious mononucleosis (IM) but could not effectively prevent EBV infection (13). It is difficult to conduct a clinical trial to evaluate the effectiveness of a prophylactic EBV vaccine in reducing the incidence of NPC or other EBV-associated cancers due to the long latency period from primary EBV infection to cancer development. However, lessons from the generation and use of vaccines against human papillomavirus (HPV) and human hepatitis B virus (HBV) indicate that the development of an effective EBV vaccine would be facilitated by the use of surrogate markers such as neutralizing antibody titer (14). Recently, studies indicated that immunization of nanoparticles displaying gp350, gH/gL, and gp42 elicited strong neutralizing antibody against EBV, showing that non-gp350 glycoproteins should also be taken into consideration for EBV vaccine design (15, 16).

In this current study, we detected the plasma neutralization ability against EBV infection of epithelial cells and B cells and the levels of IgG and IgA antibodies targeting EBV glycoproteins including gp350, gH/gL, gB, and gp42 in incident NPC cases, high-risk healthy controls (HC), and low-risk healthy controls (LC) from the screening program in Sihui County in Guangdong Province of China. Sihui is an area of NPC endemicity. The incidence rates of NPC for males and females in Sihui were about 30/10,000 and 13/100,000, respectively (17). However, NPC is rare in most parts of the world, with an incidence of <1/100,000 (18). There were two important questions that we aimed to answer. First was whether high levels of antibody targeting EBV glycoproteins involved in viral entry correlated with protection against NPC in EBV-infected adults. The second is to find which glycoproteins are promising targets for EBV prophylactic vaccine design to prevent EBV infection according to the neutralization probability raised by the antigen.

## RESULTS

In order to find out whether high levels of antibodies against the major EBV glycoproteins are correlated with protection against NPC, we selected 20 NPC cases, 20 HC, and 20 LC for evaluation. There was a similar distribution of age and sex among these three groups (Table 1). We evaluated the VCA IgA levels, EBNA1 IgA levels, and *P* values of plasma obtained at the blood sampling date. Significantly higher levels of VCA IgA and EBNA1 IgA and *P* values were observed in NPC and HC groups in comparison to the LC group (see Fig. S1 in the supplemental material).

**TABLE 1:** Characteristics of study participants^*a*^

Characteristic	*n* (%)		
	NPC	HC	LC
Age (yr)			
<53	10 (50.0)	7 (35.0)	11 (55.0)
≥53	10 (50.0)	13 (65.0)	9 (45.0)
Sex			
Male	12 (60.0)	9 (45.0)	10 (50.0)
Female	8 (40.0)	11 (55.0)	10 (50.0)

a Abbreviations: NPC, nasopharyngeal carcinoma; HC, high-risk healthy controls; LC, low-risk healthy controls.

### Individuals who developed NPC have similar neutralizing activity to inhibit EBV infection of B cells and epithelial cells as healthy controls.

**FIG 1:**
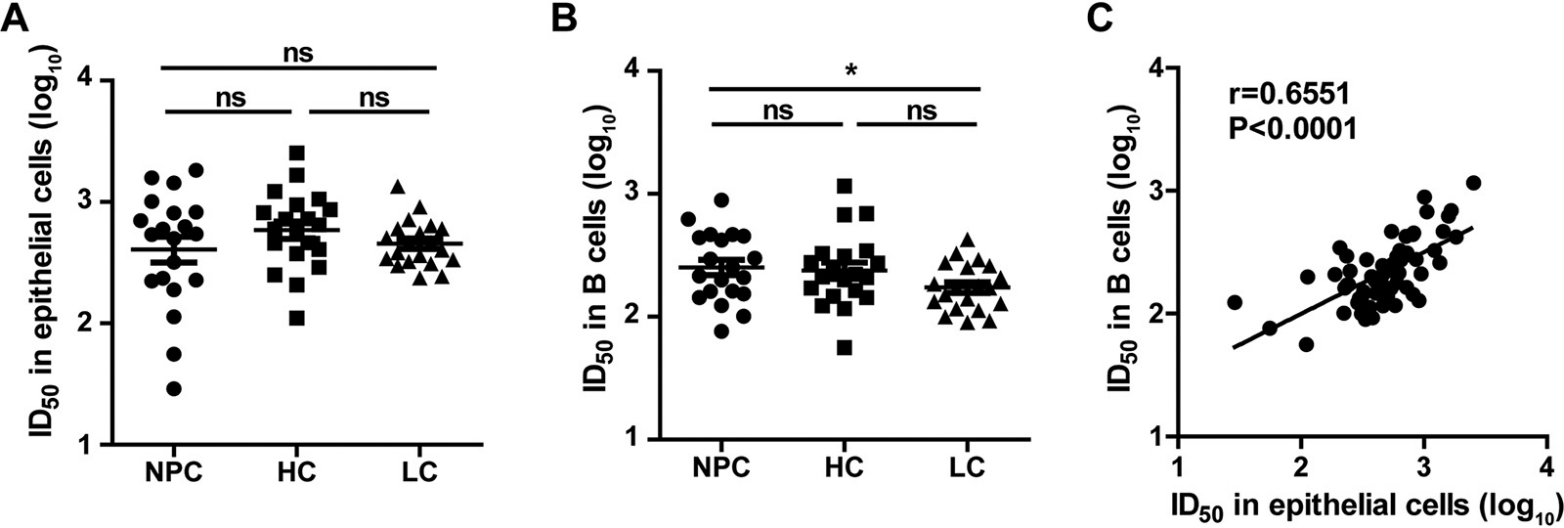
Plasma neutralization titers of nasopharyngeal carcinoma cases and control groups. (A) Neutralizing ability against Epstein-Barr virus (EBV) infection of epithelial cells represented by 50% inhibitory dilution (ID_50_) in nasopharyngeal carcinoma (NPC) cases, high-risk healthy controls (HC), and low-risk healthy controls (LC). (B) Neutralizing ability (ID_50_) against EBV infection of B cells in NPC cases, HC, and LC. (C) Correlation between the neutralizing ability (ID_50_) against EBV infection of B cells and that of epithelial cells. For definitions of significance symbols for all figures, see Materials and Methods.

Using the green fluorescent protein (GFP)-based neutralization assay, we detected the neutralizing activity of plasma samples. Neutralization against EBV infection of epithelial cells was similar among NPC, HC, and LC groups (Fig. 1A). We found that the mean 50% inhibitory dilutions (ID_50_) of B cells for NPC (307.3) and HC (303.0) were higher than that for LC (190.1), but only the difference between NPC and LC was statistically significant (*P *= 0.0244, Fig. 1B). Interestingly, with the plasma from all the groups, the neutralizing ability for B cells was significantly correlated with that for epithelial cells (Pearson = 0.6551, *P* < 0.0001) (Fig. 1C).

### Individuals who developed NPC have similar levels of IgG/IgA-specific EBV glycoprotein antibodies as low-risk healthy controls.

Levels of IgG and IgA antibody against EBV glycoproteins were measured by enzyme-linked immunosorbent assay (ELISA) using the ectodomains of major glycoproteins including gp350, gH/gL, gB, and gp42 that we purified from the corresponding plasmid-transfected 293F cells (Fig. S2A). The antigenicity of EBV glycoproteins was confirmed by detecting their binding with murine monoclonal antibody (Fig. S2B).

**FIG 2:**
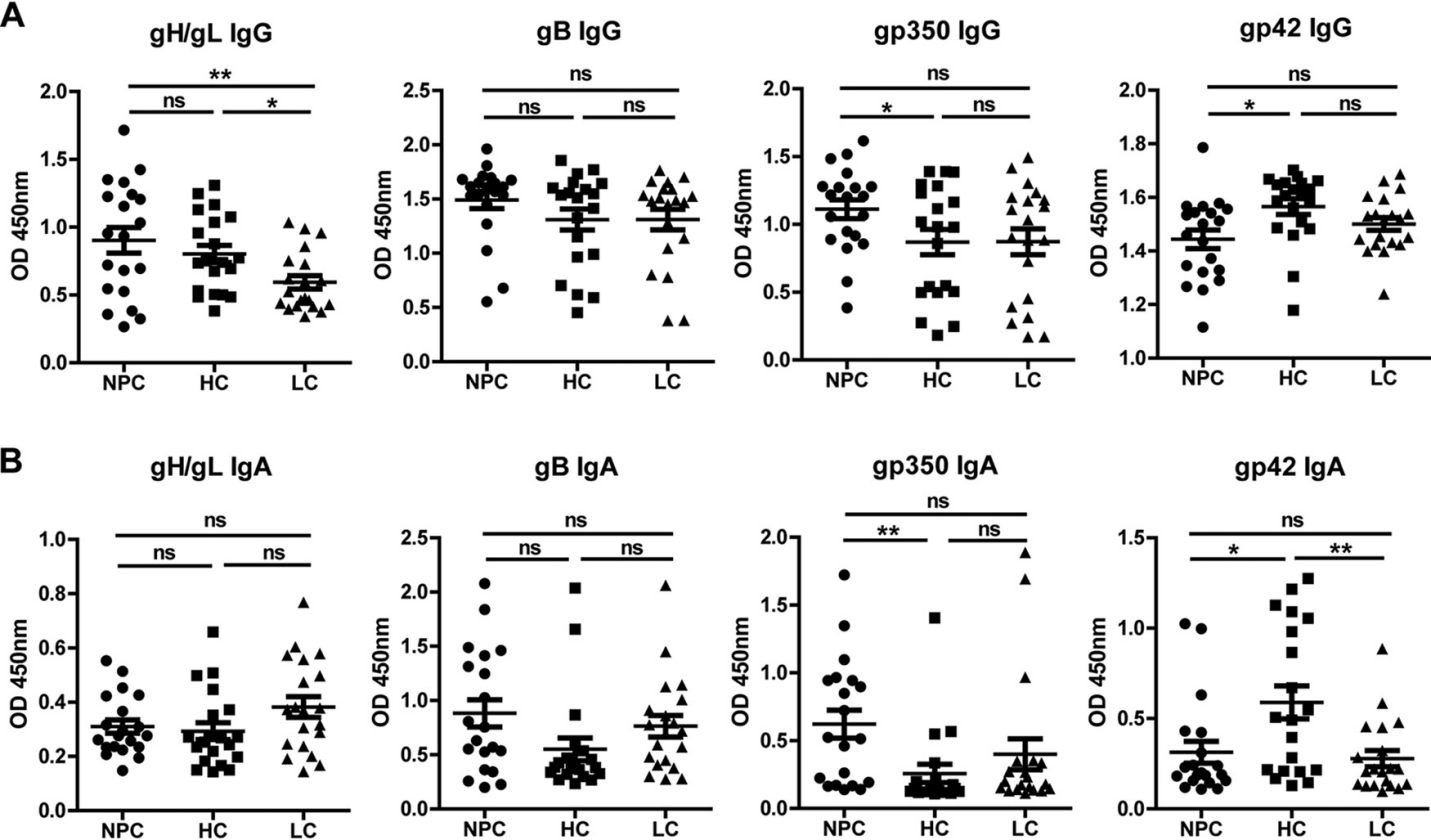
Glycoprotein antibody levels of nasopharyngeal carcinoma cases and control groups. (A) Analysis of the IgG-specific antibody levels targeting Epstein-Barr virus (EBV) glycoproteins in nasopharyngeal carcinoma (NPC) cases, high-risk healthy controls (HC), and low-risk healthy controls (LC). (B) Analysis of the IgA-specific antibody levels targeting EBV glycoproteins in NPC cases, HC, and LC.

Unlike the results reported previously (10), our data showed that the titers of IgG- and IgA-specific antibody targeting gp350 in NPC were higher than those of HC (*P* = 0.0442 and *P* = 0.0052, respectively). Interestingly, plasma levels of gp42 IgG or gp42 IgA in HC were highest among these three groups, and no significant difference was observed between NPC and LC (Fig. 2). In addition, the level of IgG-specific antibody targeting gH/gL was significantly higher in NPC and HC than in LC. However, the levels of gB IgG, gH/gL IgA, and gB IgA in these groups showed no difference (Fig. 2).

### Levels of gH/gL IgG and gB IgG were significantly correlated with the ability of participant plasma to neutralize EBV infection of epithelial cells and B cells.

**FIG 3:**
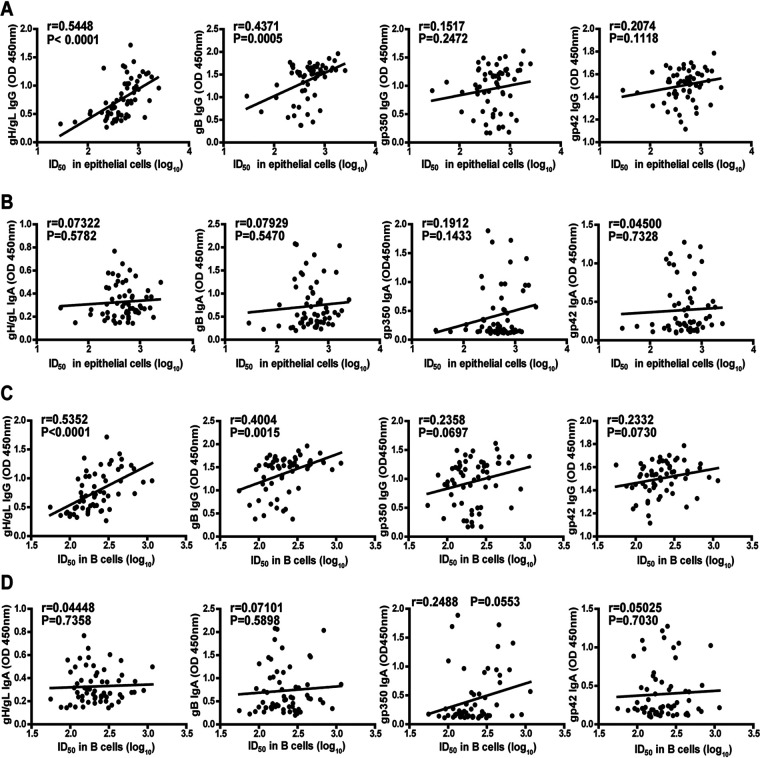
Correlation between plasma neutralization titers and glycoprotein antibody levels. (A) Correlation between neutralizing ability against Epstein-Barr virus (EBV) infection of epithelial cells represented by 50% inhibitory dilution (ID_50_) and the IgG response against gH/gL, gB, gp350, and gp42. (B) Correlation between neutralizing ability against EBV infection of epithelial cells (ID_50_) and the IgA response against gH/gL, gB, gp350, and gp42. (C) Correlation between neutralizing ability against EBV infection of B cells (ID_50_) and the IgG response against gH/gL, gB, gp350, and gp42. (D) Correlation between neutralizing ability against EBV infection of B cells (ID_50_) and the IgA response against gH/gL, gB, gp350, and gp42.

To further investigate whether levels of antibody against EBV glycoproteins could reflect the ability of neutralization against EBV infection, first, we analyzed the correlation between IgG- and IgA-specific antibody targeting gp350, gH/gL, gB, and gp42 and the neutralizing activity in epithelial cells. We found that the neutralizing ability against EBV infection of epithelial cells was in highly significant correlation with the levels of gH/gL IgG (Pearson = 0.5448, *P* < 0.0001) and gB IgG (Pearson = 0.4371, *P* = 0.0005, Fig. 3A). In general, IgG antibody was more strongly correlated with the neutralization rate in epithelial cell than was IgA antibody (Fig. 3B).

Similarly, we also analyzed the correlation between antibody targeting EBV glycoproteins and the neutralizing ability against EBV infection of B cells. Similar results showing that the neutralizing ability against EBV infection of B cells was significantly correlated with the levels of gH/gL IgG (Pearson = 0.5352, *P* < 0.0001) and gB IgG (Pearson = 0.4004, *P* = 0.0015, Fig. 3C) were observed. Likewise, IgG antibody was more strongly correlated with the neutralization rate in B cells than was IgA antibody (Fig. 3D). By measuring the viral loads of EBV-infected B cells and epithelial cells, we found that the viral loads in both cell types were significantly correlated with the levels of gH/gL IgG and gB IgG, which further confirmed the GFP-based neutralizing results (Fig. S3).

## DISCUSSION

Our study represented the first comprehensive evaluation of the association between levels of IgG and IgA antibody targeting major EBV glycoproteins involved in viral entry, including gp350, gH/gL, gB, and gp42, and neutralizing ability against EBV infection of epithelial cells and B cells and the risk of NPC. Plasma samples of NPC, HC, and LC that were collected from the NPC screening program were important for us to better elucidate whether high levels of antibodies against EBV glycoproteins were protective biomarkers for NPC.

In this study, we observed similar neutralizing ability against EBV B-cell infection in NPC cases as in the healthy controls, which was consistent with the result found in a general population cohort in Taiwan (11). In order to avoid the potential reverse causality resulting from higher EBV immune response in those with existing but undiagnosed cancer, we also detected the neutralizing ability of plasma samples collected >2 years before diagnosis and observed no significant between-sample change stratified by time of enrollment and NPC diagnosis (>2 years versus 1 to 2 years) (data not shown). In contrast, a cohort from multiplex families in Taiwan showed that individuals who develop NPC have reduced levels of neutralizing antibody that prevents EBV infection of B cells (10). The difference may be largely due to the study population. It has been reported that immune response against EBV in the multiplex family differed from that in the general population (19). It has been demonstrated that humoral immune response plays an important role in controlling primary EBV infection (20). Increased levels of EBV-neutralizing antibody, including antibody targeting gp350 and gp42, were observed over time after primary EBV infection in a cohort of university students and were associated with control of EBV infection. However, it has been thought that the cellular immune response to EBV was more critical to control severe EBV-related diseases than antibody response because of its ability to recognize and eliminate EBV-infected cells and thus limit the outgrowth of EBV-transformed cells (21). Persons with impaired T-cell immunity, such as patients with congenital immunodeficiency and transplant recipients, are more likely to develop severe EBV diseases, which provide the convincing evidence to prove the cellular immunity role (22, 23). As for NPC, EBV in the tumor cells is mainly in latency II, which is its predominant state in long-term infection, and only latency proteins like EBNA1 and latent membrane proteins 1 and 2 (LMP1 and -2, respectively) are expressed without glycoproteins (13). Therefore, neutralizing antibody could not effectively eliminate EBV-infected cells, which could partially explain why high levels of neutralizing ability detected in NPC cases were not protective for NPC development.

Previous study found that IgA-specific antibody against gp350 was evaluated close to the time of NPC diagnosis using a luciferase immunoprecipitation (LIPS) assay, but no difference was observed in the total gp350 antibody level (11). In this study, we used proteins produced by 293F cells to establish an ELISA for detecting IgG and IgA antibodies against EBV glycoproteins. Levels of gp350 IgG and gp350 IgA were significantly elevated in NPC cases compared to HC. It is possible that the small size of the study population and the different detection method led to the inconsistent results. Except for anti-gp350 antibody, we also detected the levels of antibodies to gH/gL, gB, and gp42. It is noteworthy that both IgG and IgA antibody levels against gp42 were significantly elevated in HC in comparison to NPC cases. These results suggest that anti-gp42 antibodies may be the predictive biomarkers to distinguish NPC cases from high-risk populations. However, as the sample size of 20 per group was small, we would further investigate the prediction effect of gp42 antibody in a future study with larger sample sizes. In addition, the mean IC_50_ of B cells for HC was similar to that for NPC cases. This may indicate that the correlation between gp42-IgG/IgA and neutralizing ability in B cells was not significant, suggesting that anti-gp42 antibodies did not contribute much to the plasma neutralization in this cohort. Therefore, anti-gp42 antibodies were more likely to be predictive biomarkers rather than protective biomarkers for distinguishing NPC patients from high-risk asymptomatic persons. Moreover, our data showed that levels of IgG antibody against glycoproteins were higher than those of IgA antibody. This was in line with previous findings (24), which indicated that more frequent IgG responses rather than IgA responses against glycoproteins were mounted in EBV-infected adults.

Recently, an anti-gH/gL monoclonal antibody named AMMO1, which was isolated from memory B cells of an EBV-seropositive donor, could strongly neutralize EBV infection in both epithelial cells and B cells (25). And in the humanized mouse model, AMMO1 showed excellent neutralizing ability against EBV infection of lymphocyte populations. However, 72A1, which was thought to be a neutralizing antibody targeting gp350, failed to prevent EBV-driven changes and reduce the viral load in humanized mice (26). Bu et al. found that antibodies to EBV gH/gL play an important role in epithelial-cell and B-cell-neutralizing titers against EBV infection in human plasma (16). Consistent with these studies, we observed highly significant correlations between gH/gL IgG antibody and neutralizing ability against EBV infection of epithelial cells and B cells. In addition, our data showed that IgG-specific antibody against gB was also important to prevent EBV infection of both cell types. But unlike the previous studies (27), we could not find a statistically significant association between gp350 IgG and gp42 IgG and the neutralizing ability. The inconsistency may be due to different study populations. Moreover, our results might indicate that the antibodies elicited by EBV fusion apparatus glycoproteins gH/gL and gB are more important to prevent primary EBV infection than those induced by gp350 and gp42. It has been reported that the gp350 vaccines could reduce the occurrence of IM but could not effectively prevent EBV infection (13). These results may be caused by the low titer of neutralizing antibodies induced by the gp350 vaccine alone, which cannot completely block the viral infection, and by the need to generate a local mucosal immune response to block the initial infection. Therefore, strategies of vaccine improvement through either improving the adjuvants or incorporating protein polymers such as virus-like particle (VLP) or nanoparticles were evaluated (28–33). Moreover, developing a vaccine for the combination of gH/gL and gB may increase the diversity of neutralizing antibodies to prevent EBV infection (16).

The glycoproteins and VCA are conserved among different EBV strains (34, 35), indicating that the differences of these proteins between the EBV strains in the subjects and the Akata strain were small and that our ELISAs produced in-house were suitable for detecting the levels of antibody against EBV glycoproteins in different individuals. Although the conservation of EBNA1 of different EBV strains was not as high as that of glycoproteins (36, 37), the EBNA1 IgA ELISA kit used in this study, which measured antibody levels using the antigen fragments, was approved by the Chinese Food and Drug Administration for marketing when the mass NPC screening was initiated. Therefore, it was thought that different strains in the subjects have little effect on the detection of EBNA1 IgA.

In this study, each sample was available for a long-term follow-up assessment to observe the disease progression. The sample size was limited due to the low number of incident NPC cases. However, the magnitude of the association observed was large, and the consistent data from separate assays measuring plasma neutralization against EBV infection and levels of glycoprotein antibody increased confidence in our conclusions. Similar limitations could be found in some other studies (10, 11). Further investigations with larger sample sizes and other cohorts from both areas of endemicity and areas of nonendemicity are warranted.

In conclusion, we evaluated the association between antibody responses to EBV glycoproteins, neutralization of infectivity, and the risk of NPC. We found that high levels of neutralizing antibody against EBV glycoproteins were not protective biomarkers for NPC development. Instead, they may just reflect the EBV replication and immune response in EBV-infected individuals (20). More study is needed to explore whether the T-cell reactivity to EBV proteins was responsible for preventing the tumorigenesis of NPC (38, 39). EBV gp350 protein has been the primary target of EBV vaccine in past studies. However, our results suggested that other glycoproteins, gH/gL and gB, are more important for EBV vaccine design. Neutralizing antibodies induced by these glycoproteins may provide more comprehensive protection against primary EBV infection for the EBV-seronegative populations. But for the long-term EBV-infected adults from high-NPC-incidence region, EBV vaccine that could induce T-cell immunity may be more effective to prevent the occurrence and development of NPC.

## MATERIALS AND METHODS

### Ethical statement.

This study was reviewed and approved by the Ethics Committee of the Sun Yat-Sen University Cancer Center (SYSUCC; Guangzhou, Guangdong, China) and was conducted in accordance with the Declaration of Helsinki.

### Study population.

To evaluate the correlation between EBV glycoprotein IgG and IgA antibody levels and the neutralizing ability of plasma, we collected stratified samples of 60 participants including 20 histologically diagnosed NPC cases, 20 non-NPC high-risk healthy controls (HC), and 20 low-risk healthy controls (LC) in a screening program in Sihui County in Guangdong Province of China from 2007 and 2018. Individuals in the three groups were chosen with similar gender and age distributions. All NPC samples were obtained before cancer diagnosis. Among NPC cases, 19 plasma samples were obtained within 1 to 2 years and one was over 2 years before diagnosis. And all patients did not receive treatment for NPC before plasma collection. Plasma samples from HC and LC were chosen at the last follow-up. The screening program has been introduced in detail in other papers (5, 40). The high-risk and low-risk individuals were defined from 2 screening markers of EBV VCA/IgA and EBNA1/IgA antibodies. These two markers were selected as highly predictive of NPC risk based on a previous work, and a predefined algorithm (LogitP = −3.934 + 2.203 × VCA/IgA + 4.797 × EBNA1/IgA) that combined results from the two tests was used to calculate an EBV-based risk score (P) for NPC (5, 40). Using this combined score, individuals defined as positive (*P* ≥ 0.98) were defined as high-risk individuals, while those with a *P* value of <0.65 were defined as the low-risk group. High-risk and low-risk individuals had different NPC risks and were repeatedly tested for EBV antibodies with different intervals. This mass screening study was approved by the Ethics Review Committee of the Sun Yat-sen University Cancer Center.

### EBV neutralization assay.

The neutralization assay was conducted using type I EBV (Akata strain), which was prepared as previously described (41). Plasma samples from study individuals were serially diluted in 3-fold steps (from 1:20 to 6 serial dilutions) and incubated with green fluorescent protein (GFP)-expressing EBV for 2 h at 37°C. Then, the mixtures were added to Raji B cells or HK1 epithelial cells and incubated for 3 h at 37°C. Then, the unbound virus was removed and infected cells were cultured in fresh medium for 48 h, followed by detection of GFP-positive cells using a flow cytometer (Beckman cytoFLEX). Neutralization rate of each plasma sample was defined as: (% GFP^+^ cells in the positive-control well containing virus alone − % GFP^+^ cells in the plasma-containing well)/% GFP^+^ cells in the positive-control well × 100. Fifty percent inhibitory dilution (ID_50_) was calculated by GraphPad Prism 8.0 software. Neutralization assay of B cells or epithelial cells was done in 1 day. Positive control was performed in duplicate wells. The 100% infectivity ranges of B cells and epithelial cells were 40.58% to 41.77% and 7.01% to 7.12%, respectively. And the typical % GFP positivity was 20.58% and 3.53%, respectively, to which ID_50_ of B cells and epithelial cells were normalized. In order to measure the viral loads in EBV-infected epithelial and B cells, 19 plasma samples from NPC cases and high-risk and low-risk controls (6 to 7 per group) were diluted at 1:300 and incubated with EBV as described above. Forty-eight hours later, the DNA of Raji cells and HK1 cells was extracted using a DNA kit (Omega). EBV DNA was detected by real-time PCR using the TaqMan BamHI probes as previously described (7). Relative viral load was determined using glyceraldehyde-3-phosphate dehydrogenase (GAPDH) as an internal control.

### Expression and purification of recombinant proteins.

The ectodomains of gp350, gB, gH/gL, and gp42 fragments were amplified from the bacterial artificial chromosome of the EBV-M81 strain and cloned into pcDNA3.1 plasmid with an N-terminal CD5 leader peptide and a C-terminal His tag. Plasmids were transfected into 293F cells. After 5 days, cell supernatant was collected and passed through nickel-nitrilotriacetic acid (Ni-NTA) resin, followed by washing (phosphate-buffered saline [PBS] with 20 mM imidazole, pH 7.4) and elution (PBS with 250 mM imidazole, pH 7.4). Proteins were further purified by size exclusion chromatography (SEC) and dialyzed into PBS.

### EBV antibody assays.

Anti-VCA IgA and anti-EBNA1 IgA were detected by commercial kits as previously described (5). One hundred nanograms/well of EBV glycoprotein was coated in 96-well enzyme-linked immunosorbent assay (ELISA) plates overnight at 4°C. Then, plates were blocked with PBS containing 5% bovine serum albumin and 0.1% Tween 20 (blocking buffer) at 37°C for 1 h. After blocking, plates were washed three times with 0.1% Tween 20 in PBS (washing buffer). Plasma samples were diluted 1:100 in blocking buffer and incubated at 37°C for 1 h. Following washing five times, a 1:2,000 dilution of goat anti-human IgA-horseradish peroxidase (HRP) (Boster Corporation) or 1:4,000 goat anti-human IgG-HRP (Abbkine) in blocking buffer was added to each well and incubated at 37°C for 45 min. Plates were washed five times and incubated with 3,3′,5,5′-tetramethylbenzidine substrate (Tiangen) for 5 min at room temperature. Then, 1 N hydrochloric acid was added and optical density at 450 nm (OD_450_) was read on a microplate reader (Bio-Tek Epoch2). The binding ability of purified murine monoclonal antibodies E1D1, CL55, 72A1, and 3H3 with corresponding EBV glycoproteins gH/gL, gB, gp350, and gp42 was also detected by the in-house-produced ELISAs. Indicated antibodies were diluted serially in blocking buffer and incubated at 37°C for 1 h. Following washing three times, 1:4,000 goat anti-mouse IgG-HRP (Invitrogen) in blocking buffer was added to each well and incubated at 37°C for 45 min. The remaining steps were the same as described above.

### Statistical analyses.

GraphPad Prism version 8.0 was used for statistical analysis. We compared the EBV glycoprotein IgG-specific and IgA-specific antibody levels and neutralization of EBV B-cell and epithelial-cell infection between two groups (i.e., NPC versus HC) using an unpaired, Welch-Satterthwaite *t* test that did not assume equal variance. We estimated the correlation between plasma neutralization against EBV infection or relative viral load of epithelial cells or B cells and the levels of IgG or IgA antibody targeting EBV glycoproteins (i.e., neutralization of B cells versus gH/gL IgG), using Pearson correlation coefficients. *P* values less than 0.05 were considered to be statistically significant (*, *P* < 0.05; **, *P* < 0.01; ***, *P* < 0.001; ns, not significant).

10.1128/mSphere.00901-20.1 FIG S1 Screening analyses of collected plasma samples from nasopharyngeal carcinoma cases and control groups. (A) Analysis of the IgA response to Epstein-Barr virus (EBV) capsid antigen (VCA) of samples from nasopharyngeal carcinoma (NPC) cases, high-risk healthy controls (HC), and low-risk healthy controls (LC). (B) Analysis of the IgA response to EBV nuclear antigen-1 (EBNA1) of samples from NPC cases, HC, and LC. (C) Analysis of LogitP (P) value of samples from NPC cases, HC, and LC. *P* = −3.934 + 2.203 × VCA/IgA + 4.797 × EBNA1/IgA. Download FIG S1, TIF file, 0.4 MB. Copyright © 2020 Zhu et al. 2020 Zhu et al. This content is distributed under the terms of the Creative Commons Attribution 4.0 International license.

10.1128/mSphere.00901-20.2 FIG S2 Purification of EBV glycoproteins. (A) Purified EBV glycoproteins were separated by reducing and nonreducing SDS-PAGE and stained with Coomassie brilliant blue. (B) The binding between murine monoclonal antibodies and EBV glycoproteins was detected by ELISA. Download FIG S2, TIF file, 2.0 MB. Copyright © 2020 Zhu et al. 2020 Zhu et al. This content is distributed under the terms of the Creative Commons Attribution 4.0 International license.

10.1128/mSphere.00901-20.3 FIG S3 Correlation between viral loads and glycoprotein antibody levels. (A) Correlation between relative viral load in epithelial cells and the IgG response against gH/gL, gB, gp350, and gp42. (B) Correlation between relative viral load in B cells and the IgG response against gH/gL, gB, gp350, and gp42. Download FIG S3, TIF file, 0.9 MB. Copyright © 2020 Zhu et al. 2020 Zhu et al. This content is distributed under the terms of the Creative Commons Attribution 4.0 International license.
