# Supplementary figures and images for: Association between Antibody Responses to Epstein-Barr Virus Glycoproteins, Neutralization of Infectivity, and the Risk of Nasopharyngeal Carcinoma
Source: mSphere. 2020 Dec 2;5(6):e00901-20. doi: 10.1128/mSphere.00901-20 (PMC7716278; doi:10.1128/mSphere.00901-20)

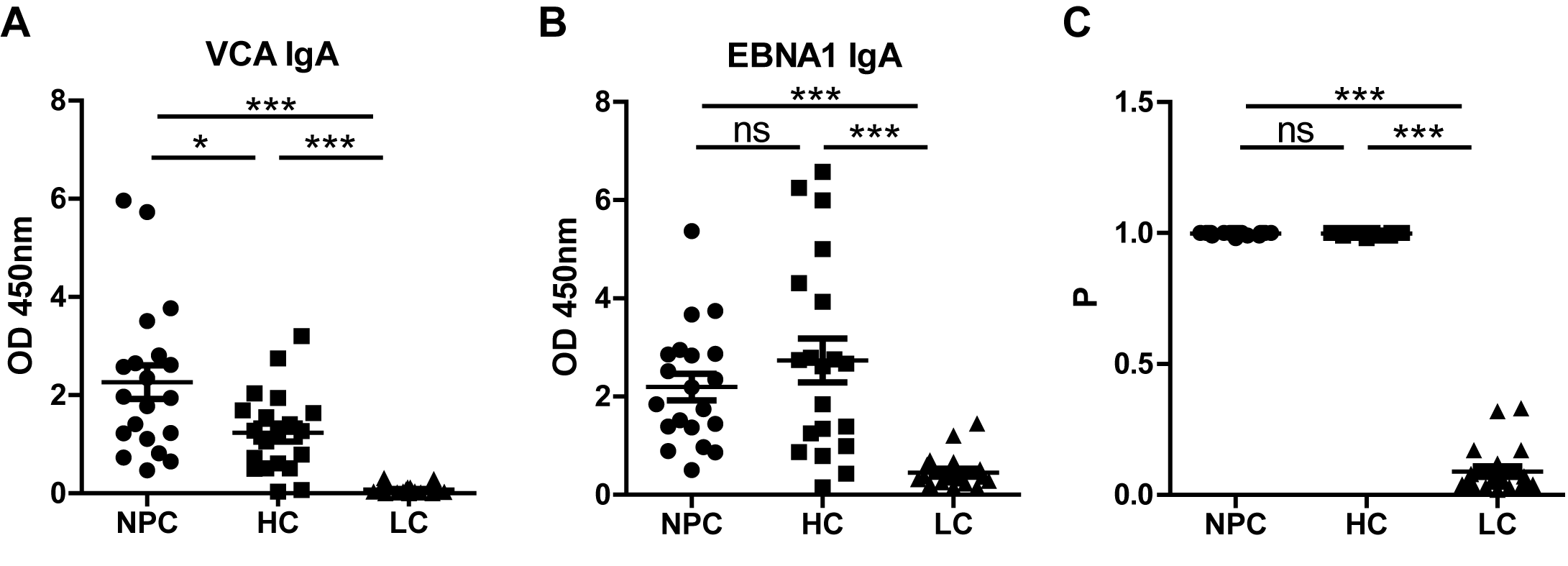

Supplement: FIG S1 [file mSphere.00901-20-sf001.tif]

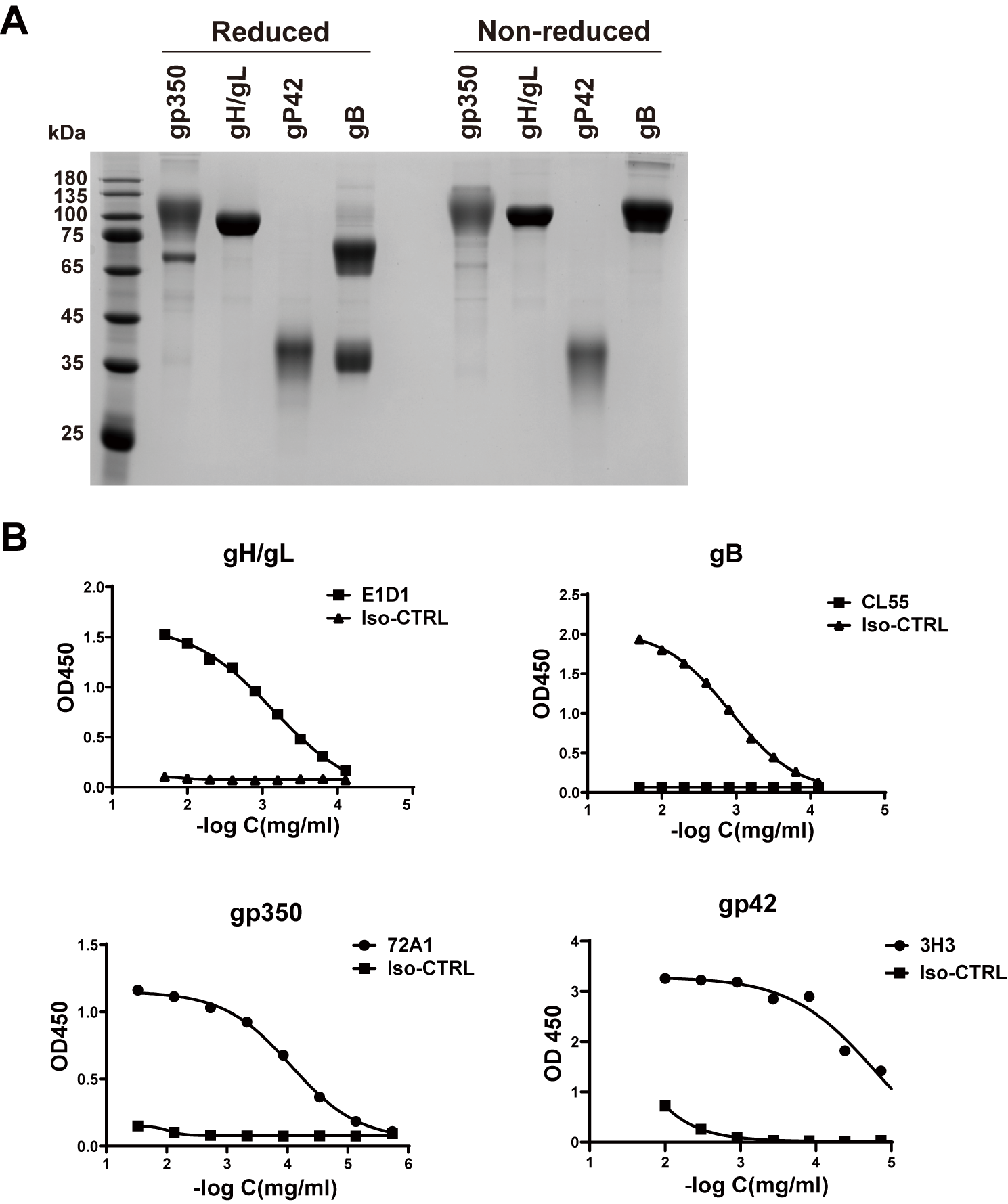

Supplement: FIG S2 [file mSphere.00901-20-sf002.tif]

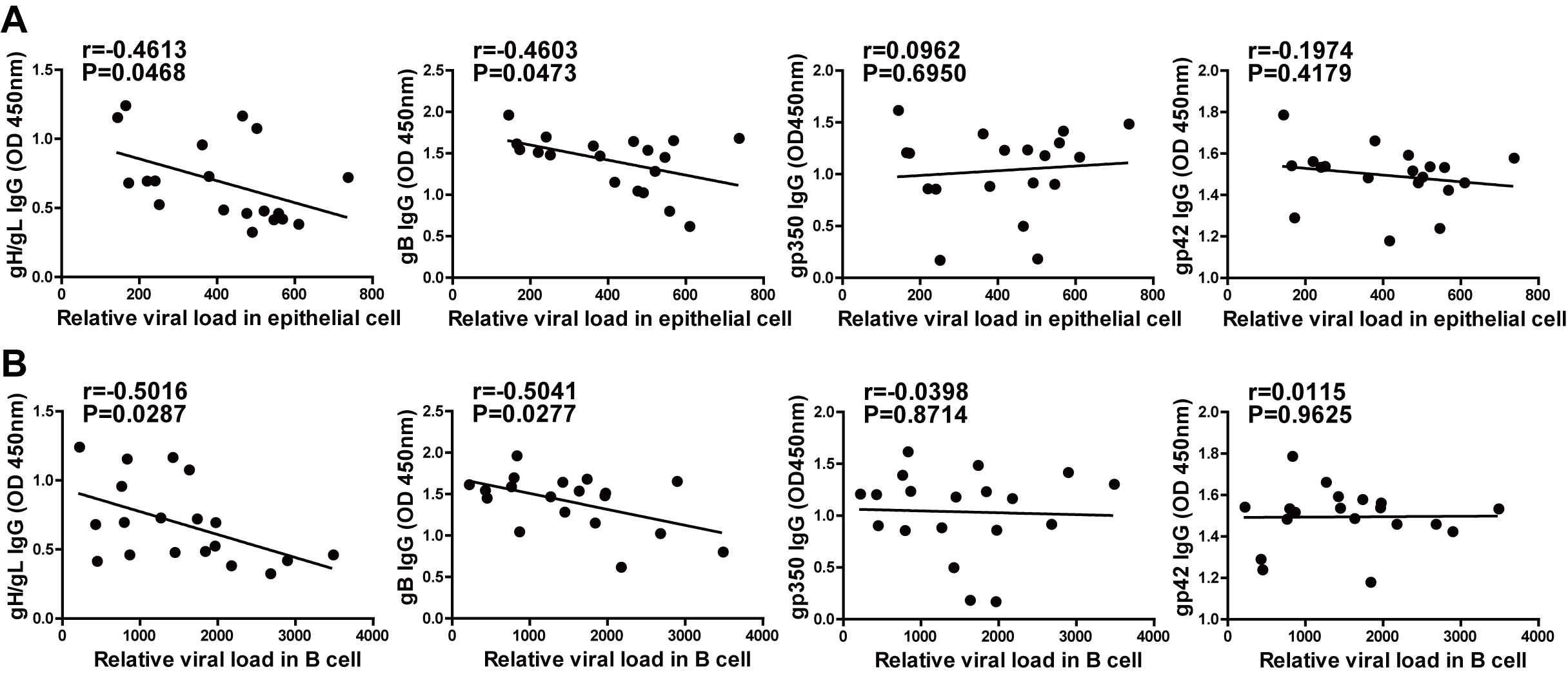

Supplement: FIG S3 [file mSphere.00901-20-sf003.tif]
